# Differences in clinical features and prognosis between hypertriglyceridemia and other causes of acute pancreatitis: a dual perspective based on metabolic disorders and inflammatory response

**DOI:** 10.3389/fendo.2026.1851662

**Published:** 2026-06-10

**Authors:** Tong Wu, Yan Zhang, Linzhen Li

**Affiliations:** Departments of Gastroenterology, First Affiliated Hospital of Wannan Medical College, Wuhu, Anhui, China

**Keywords:** blood glucose, hypertriglyceridemia acute pancreatitis, inflammatory response, metabolic disorders, uric acid

## Abstract

**Background:**

To investigate the clinical features and prognosis of acute pancreatitis (AP) with different etiologies.

**Methods:**

A total of 242 patients with AP admitted to the First Affiliated Hospital of Wannan Medical College from March 2025 to January 2026 were prospectively collected and divided into two groups according to the etiology: hypertriglyceridemia acute pancreatitis (HTG-AP) group and non-hypertriglyceridemia acute pancreatitis (non-HTG-AP) group. The study compared the metabolic characteristics, inflammation level, short-term severe rate and long-term abnormal glucose metabolism between the two groups of AP patients.

**Results:**

This prospective single-center study enrolled 242 AP patients (80 HTG-AP and 162 non-HTG-AP). Compared with non-HTG-AP patients, HTG-AP patients were significantly younger (*P* < 0.001), had higher body mass index (BMI) (*P* < 0.001) and diabetes prevalence (*P* < 0.001), but lower cardiovascular disease rate *(P* < 0.01). Fasting blood glucose, serum calcium, albumin, uric acid, lipid profiles, white blood cells, lymphocytes, neutrophils, platelets and C-reactive protein (CRP) were all significantly higher in the HTG-AP group (all *P* < 0.05), accompanied by a higher proportion of moderately severe acute pancreatitis (MSAP) and severe acute pancreatitis (SAP) (*P* < 0.05). The rate of the intensive care unit (ICU) admission was significantly higher in the HTG-AP group than in the non-HTG-AP group (*P* < 0.05). Among 172 nondiabetic patients followed for 3 months, HTG-AP was associated with a markedly higher incidence of post-acute pancreatitis diabetes mellitus (PPDM-A) (*P* < 0.05), suggesting an increased long-term risk of islet dysfunction.

**Conclusions:**

Compared with other etiologies of acute pancreatitis, HTG-AP patients present younger onset, more severe glucose, lipid, calcium and uric acid metabolic disorders, and stronger systemic inflammatory response, with higher rates of severe pancreatitis and significantly elevated long-term risk of new-onset diabetes.

## Introduction

Acute pancreatitis (AP) is a systemic disease caused by local inflammatory activation of the pancreas (1). It is a common acute and critical disease of the digestive system. Its global incidence is increasing year by year (2). The etiology of AP is complex and diverse, including biliary, alcoholic, and hypertriglyceridemia. In recent years, with the improvement of living standards and the change of dietary structure among the Chinese population, the incidence of hypertriglyceridemia acute pancreatitis (HTG-AP) has increased significantly (3). It has surpassed alcoholic pancreatitis and has become the second most common cause of AP in China, accounting for 20.0% to 32.8% of all AP (3, 4). The core essence of the pathogenesis of HTG-AP is lipotoxic injury, that is, pancreatic lipase catalyzes the hydrolysis of triglycerides to generate excessive free fatty acids, which directly mediates local pancreatic injury and systemic multiple organ damage (5). Its pathophysiological mechanism may lead to the unique characteristics of HTG-AP in clinical manifestations, complications and prognosis. At present, most of the studies on HTG-AP at home and abroad are retrospective analysis (6–8). There is a lack of evidence from prospective cohort studies. Few studies have systematically compared the baseline metabolic characteristics, inflammation level, short-term severe and long-term abnormal glucose metabolism outcomes of HTG-AP with other causes of AP. Therefore, this study aims to provide evidence-based support for precise intervention and long-term management of HTG-AP by comparing the clinical characteristics and prognosis of HTG-AP and other types of AP.

## Materials and methods

### Study population

A prospective cohort study was conducted at the First Affiliated Hospital of Wannan Medical College from March 2025 to January 2026. Participants were recruited by consecutive sampling: all patients admitted with acute pancreatitis during the study period were screened, and those who met the eligibility criteria were enrolled. The inclusion criteria were as follows: (1) patients who met the AP diagnostic criteria need to meet at least two of the three criteria according to the 2012 Revised Atlanta Classification (1): typical abdominal pain, serum lipase/amylase > 3 times the upper limit of normal, and imaging support; (2) age ≥ 18 years; (3) the time from onset to admission was ≤ 24 hours. The exclusion criteria were as follows: (1) two or more mixed causes; (2) prior diagnosis of active malignancy (excluding definitively treated early-stage cancer without recurrence within 5 years), end-stage renal disease requiring maintenance dialysis, decompensated cirrhosis, active autoimmune disease on systemic immunosuppressive therapy, or any other serious comorbidity judged by the investigator to severely affect study participation; (3) discharge or death within 24 hours of hospitalization.

### Definition

HTG-AP can be diagnosed if serum triglycerides (TG) are > 1000 mg/dL (11.3 mmol/L), or TG is between 500–1000 mg/dL (5.65-11.3 mmol/L) with chylous serum, after excluding other etiologies (4).

According to the revised Atlanta classification criteria (1), this study classified the severity of AP into two groups: mild acute pancreatitis (MAP) and non-mild acute pancreatitis (non-MAP). MAP refers to the absence of organ failure and no local or systemic complications. Non-MAP includes moderately severe acute pancreatitis (MSAP) and severe acute pancreatitis (SAP). MSAP/SAP is characterized by organ failure and local or systemic complications.

In this study, post-acute pancreatitis diabetes mellitus (PPDM-A) was defined as new-onset diabetes that met the diagnostic criteria of the American Diabetes Association (9), occurred three months after the onset of acute pancreatitis, and excluded cases with stress hyperglycemia or a prior history of diabetes. Elevated blood glucose during AP that normalized and no longer met the American Diabetes Association criteria at the three-month follow-up was classified as stress hyperglycemia and excluded.

### Data collection

This was a prospective cohort study. Eligible patients with acute pancreatitis were recruited at admission. Demographic information, BMI, medical history, laboratory and imaging findings, length of stay, and in-hospital outcomes were recorded from the medical records and hospital electronic medical record system. Participants were followed prospectively, and the occurrence of PPDM-A was assessed at 3 months after discharge.

### Statistical analysis

SPSS 27.0 software was used for statistical analysis. Continuous variables were tested for normality using the Shapiro-Wilk test. Data that followed a normal distribution are presented as mean ± standard deviation and compared using the independent t-test. Data that deviated from normality are presented as median (interquartile range) and compared using the Mann-Whitney U test. Because all continuous variables in this study were found to be non-normally distributed, they are presented as median (IQR) throughout. Categorical variables were expressed as frequency (%) and compared using the chi-squared or Fisher’s exact test. A difference at P < 0.05 was deemed to be statistically significant.

### Ethics approval and consent to participate

The research protocol complies with the Declaration of Helsinki Ethical Guidelines and was approved by the institutional review board of Wannan Medical College (2025.130). All participants provided written informed consent.

## Results

### Basic demographic features

**Figure 1:**
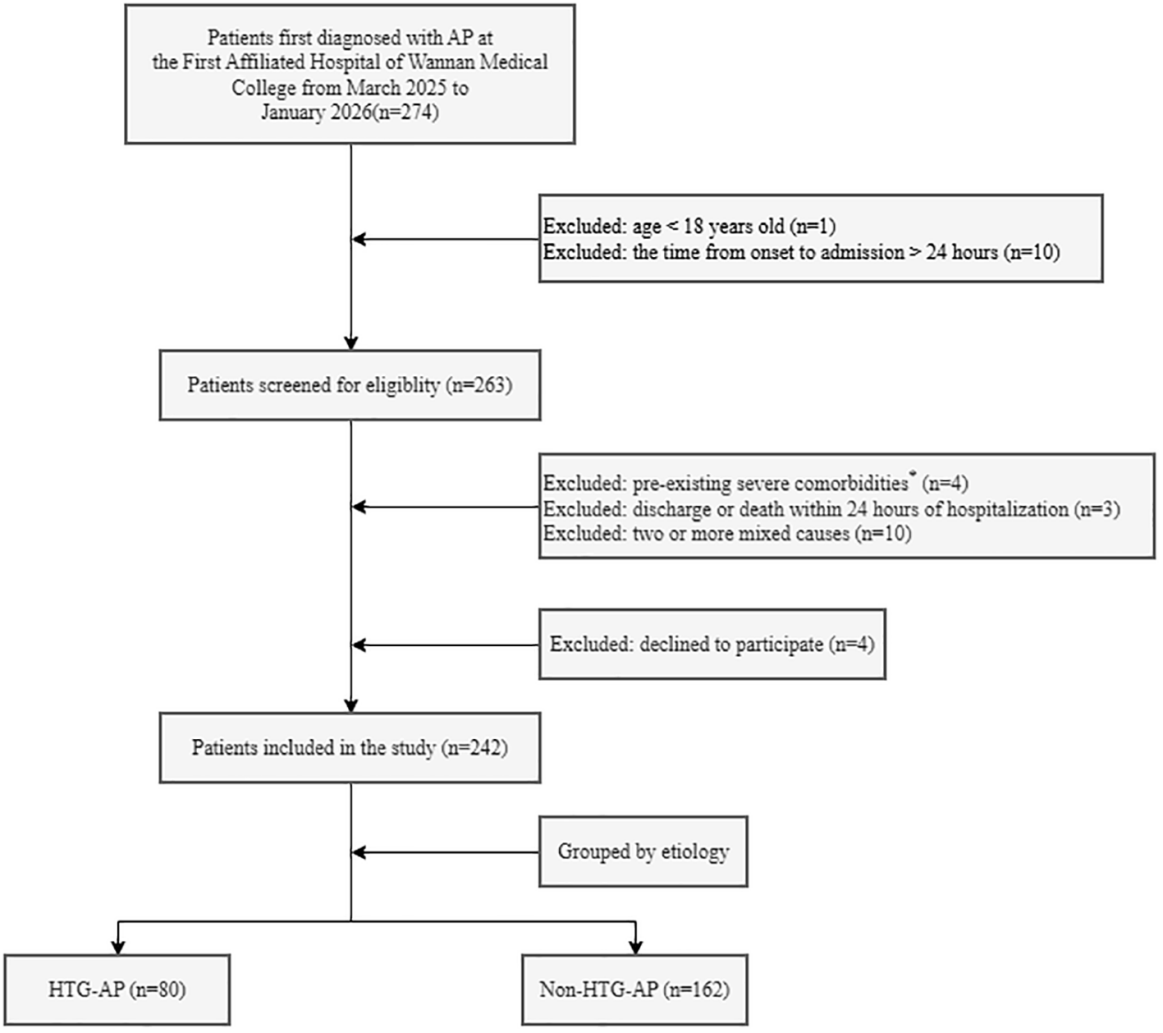
Research sample screening and grouping process. AP, acute pancreatitis; HTG-AP, hypertriglyceridemia acute pancreatitis. ^*^Defined as prior diagnosis of active malignancy (excluding definitively treated early-stage cancer without recurrence within 5 years), end-stage renal disease requiring maintenance dialysis, decompensated cirrhosis, active autoimmune disease on systemic immunosuppressive therapy, or any other serious comorbidity judged by the investigator to severely affect study participation.

In this prospective single-center study, a total of 274 patients were screened, of whom 242 eligible AP patients provided written informed consent, were consecutively enrolled, and were included in the study. All 242 completed the 3-month follow-up with no loss (**Figure 1**). Among them, 80 patients were in the HTG-AP group and 162 patients were in the non-HTG-AP group. A comparison of the demographic and clinical characteristics of the two groups is shown in **Table 1**. Compared with non-HTG-AP patients, HTG-AP patients were younger (37.5 vs 55, *P* < 0.001), had higher BMI (27.05 vs 23.93, *P* < 0.001), a higher prevalence of diabetes (52.5% vs 17.3%, *P* < 0.001), and had a lower prevalence of cardiovascular disease (3.8% vs 18.5%, *P* = 0.002). However, there was no statistically significant difference in gender (*P* = 0.745) and history of hypertension (*P* = 0.293) between the two groups of patients.

**Table 1:** Comparison of clinical characteristics and underlying conditions between the two groups.

Parameters	HTG-AP (n = 80)	non-HTG-AP (n = 162)	*P* value
Age, year	37.50 (33.00, 44.75)	55.00 (39.00, 62.00)	<0.001
Gender, male/female	55/25	108/54	0.745
BMI, kg/m^2^	27.05 (24.48, 29.09)	23.93 (21.62, 27.00)	<0.001
Hypertension, n (%)	27 (33.8%)	66 (40.7%)	0.293
Diabetes, n (%)	42 (52.5%)	28 (17.3%)	<0.001
Cardiovascular disease, n (%)	3 (3.8%)	30 (18.5%)	0.002

HTG-AP, hypertriglyceridemia acute pancreatitis; non-HTG-AP, non-hypertriglyceridemia acute pancreatitis; BMI, body mass index.

### Laboratory data and severity of the disease

The comparison of the laboratory indicators between the two groups within 24 hours of admission is shown in **Table 2**. The levels of fasting blood glucose, serum calcium, albumin, uric acid (UA), total cholesterol (TC), triglycerides (TG), high-density lipoprotein cholesterol (HDL-C), and low-density lipoprotein cholesterol (LDL-C) in the HTG-AP group were significantly higher than those in the non-HTG-AP group (all *P* < 0.05). The levels of white blood cells (WBC), neutrophils, lymphocytes, platelets (PLT) and C-reactive protein (CRP) in HTG-AP group were also significantly higher than those in non-HTG-AP group (all *P* < 0.05). Regarding disease severity, the proportion of severe pancreatitis (MSAP/SAP) was significantly higher in the HTG-AP group (*P* = 0.015). The rate of the intensive care unit (ICU) admission was significantly higher in the HTG-AP group than in the non-HTG-AP group (*P* = 0.029). However, there was no significant difference in hospital stay between the two groups, which may be related to the sample size.

**Table 2:** Comparison of serological-related indicators and disease severity between the two groups.

Parameters	HTG-AP (n = 80)	non-HTG-AP (n = 162)	*P* value
Fasting blood glucose, mmol/L	11.64 (8.21, 15.77)	6.27 (5.35, 8.10)	<0.001
White blood cells, *10^9^/L	11.90 (9.20, 13.90)	8.70 (6.80, 11.33)	<0.001
Neutrophils, *10^9^/L	9.25 (7.40,11.60)	7.10 (4.80, 9.50)	<0.001
Lymphocytes, *10^9^/L	1.40 (0.93, 1.80)	1.20 (0.80, 1.50)	0.043
Platelets, *10^9^/L	215.00 (170.00,260.75)	186.50 (148.00,227.75)	0.007
CRP, mg/L	40.15 (9.89, 154.38)	15.60 (3.92, 63.42)	<0.001
Serum calcium, mmol/L	2.19 (2.06, 2.36)	2.11 (2.00, 2.20)	0.001
Albumin, g/L	41.25 (39.60, 44.35)	39.60 (37.05, 42.33)	<0.001
Uric acid, umol/L	352.75 (277.00, 431.70)	289.65(221.10, 369.28)	<0.001
Total cholesterol, mmol/L	9.67 (7.36, 12.31)	4.34 (3.32, 5.04)	<0.001
Triglycerides, mmol/L	19.46 (13.47, 29.23)	1.20 (0.77, 1.89)	<0.001
HDL-C, mmol/L	2.30 (1.49, 3.24)	1.12 (0.94, 1.31)	<0.001
LDL-C, mmol/L	4.66 (3.73, 6.58)	2.64 (2.04, 3.34)	<0.001
MSAP/SAP, n (%)	26 (32.5%)	30 (18.5%)	0.015
Hospital stay, day	8.00 (7.00, 11.00)	8.00 (6.00, 11.00)	0.334
ICU admission, n (%)	6 (7.5%)	2 (1.2%)	0.029

HTG-AP, hypertriglyceridemia acute pancreatitis; non-HTG-AP, non-hypertriglyceridemia acute pancreatitis; CRP, C-reactive protein; HDL-C, high-density lipoprotein cholesterol; LDL-C, low-density lipoprotein cholesterol; MSAP/SAP, moderately severe acute pancreatitis/severe acute pancreatitis; intensive care unit, ICU.

### Incidence of PPDM-A

A total of 172 patients with no history of diabetes were followed up for 3 months after discharge. There were 38 patients in the HTG-AP group and 134 patients in the non-HTG-AP group. No cases were lost during the follow-up period. The proportion of PPDM-A in the HTG-AP group was significantly higher than that in the non-HTG-AP group (*P* = 0.004) (**Table 3**), suggesting that HTG-AP is not only more severe in the acute phase, but also has a higher risk of long-term islet dysfunction.

**Table 3:** Comparison of new-onset diabetes between the two groups.

Parameters	HTG-AP (n = 80)	Non-HTG-AP (n = 162)	*P* value
Follow-up cases	38	134	
PPDM-A, n (%)	10 (26.3%)	10 (7.5%)	0.004

HTG-AP, hypertriglyceridemia acute pancreatitis; non-HTG-AP, non-hypertriglyceridemia acute pancreatitis; PPDM-A, post-acute pancreatitis diabetes mellitus.

## Discussion

Acute pancreatitis is a common digestive system emergency with complex etiology. Hypertriglyceridemia has become one of the main causes of AP, and the incidence of HTG-AP is increasing year by year (10). In this study, a prospective cohort design was used to evaluate whether there were significant differences in clinical characteristics and prognosis between HTG-AP and AP caused by other causes.

The research showed that there were more males than females in AP patients. The median age and interquartile range of HTG-AP group were 37.50 (33.00, 44.75) years, and those of non-HTG-AP group were 55.00 (39.00, 62.00) years. It can be seen that HTG-AP patients are younger and exhibit a clear trend toward a younger age. This is consistent with previous research findings (3, 11). BMI is a commonly indicator to evaluate the degree of obesity. Previous studies have confirmed that elevated BMI and hypertriglyceridemia are risk factors for pancreatitis (12). Our study further found that the BMI of patients with HTG-AP was significantly higher than that of non-HTG-AP, further confirming overweight or obesity as one of the key clinical features of HTG-AP. HTG-AP patients are often accompanied by a variety of metabolic diseases, but the incidence of cardiovascular disease in the HTG-AP group is only 3%, which is significantly lower than that in the non-HTG-AP group. This difference may be mainly related to the younger age of HTG-AP patients.

There are significant metabolic disorders in HTG-AP patients. The core pathological basis of HTG-AP is severe hypertriglyceridemia (13). In this study, the median serum TG level in the HTG-AP group was approximately 16-fold higher than that in the non-HTG-AP group (19.46 vs. 1.20 mmol/L), consistent with the lipid profile characteristics of HTG-AP. Meanwhile, levels of TC, HDL-C, and LDL-C were also higher in the HTG-AP group, suggesting that most of these patients had mixed dyslipidemia. Secondly, HTG-AP patients exhibited extensive disturbances in glucose and UA metabolism. The prevalence of diabetes in HTG-AP was as high as 52.50%. Epidemiological evidence shows that patients with type 2 diabetes have a higher risk of AP than those without diabetes (14, 15). Hyperglycemia is a common early feature of AP, with an incidence of more than 30%, and it is particularly prevalent in severe patients (8, 16). In this study, the first fasting blood glucose level in the HTG-AP group was significantly higher than that in the non-HTG-AP group, and the incidence of PPDM-A was also significantly increased, suggesting that HTG-AP has a more severe impairment to pancreatic endocrine function. Liu et al. (8). found that patients with TG ≥ 5.65 mmol/L had a 3.964-fold higher risk of developing hyperglycemia compared with those with normal TG (95% CI: 1.990-7.895, *P* < 0.001), further confirming the effect of hyperlipidemia on blood glucose levels. Another point of view is that patients with metabolic syndrome combined with obesity, hyperglycemia and hyperlipidemia have an increased risk of PPDM-A in the later stage (17). These metabolic abnormalities are closely related to HTG-AP. Two meta-analyses published in 2014 and 2019 (18, 19) showed that approximately 23% of individuals developed PPDM-A after the onset of AP. Abnormal blood glucose fluctuations are closely associated with the development of PPDM. Another meta-analysis suggests that stress-induced blood glucose levels are positively associated with the risk of new-onset diabetes in patients with acute or critical illness (20). The pathophysiological relationship between HTG-AP and diabetes seems to be bidirectional. A review published in Cardiovascular Endocrinology & Metabolism in 2025 proposed the “metabolic cascade hypothesis” (21): dysfunction of adipose tissue (Adipo IR) causes the body to release a large amount of free fatty acids to the liver, inducing hepatic insulin resistance (Hep-IR); in turn, the impaired liver aggravates hyperglycemia and triglyceride synthesis. Conversely, hyperglycemia and insulin resistance further promote adipose tissue lipolysis and hepatic very-low-density lipoprotein (VLDL) secretion (22). This self-amplifying vicious cycle ultimately leads to systemic glucose and lipid metabolism disorders.

UA is the final product of purine metabolism in humans. In recent years, a number of studies have shown that high serum UA levels are associated with the development of various diseases (23–25). A prospective study (26) has identified a significant association between abnormal UA metabolism and pancreatic diseases. UA is an independent risk factor for AP (25), yet few studies have directly compared serum UA levels in patients with HTG-AP and non-HTG-AP. Our previous studies have confirmed that the serum UA concentration in patients with AP is significantly higher than that in healthy people, and the serum UA level in patients with AP showed a low linear correlation with TG levels (27). Therefore, serum UA levels may also be associated with HTG-AP. Our study found that serum UA levels in patients with HTG-AP were significantly different from those in non-HTG-AP, which may be caused by a common metabolic basis.

In addition to metabolic disorders, HTG-AP patients often show more severe systemic inflammatory response. Compared with non-HTG-AP patients, WBC, neutrophils, lymphocytes and CRP levels were significantly increased in HTG-AP patients. Recent animal model experiments have confirmed that hypertriglyceridemia can exacerbate pancreatic tissue injury in the setting of AP (28). Hyperlipidemia is not only a metabolic disorder, but also a chronic inflammatory condition. Adipose tissue itself can secrete tumor necrosis factor-α (TNF-α), interleukin-6 (IL-6) and other inflammatory factors (29). In patients with HTG-AP, high concentrations of TG and free fatty acids activate macrophages and neutrophils, leading to the release of large amounts of pro-inflammatory cytokines. This induces systemic inflammatory response syndrome and subsequently aggravates pancreatic injury (30). A retrospective study by Liu et al. (31) confirmed that the systemic inflammatory response index (SIRI) level in the HTG-AP group was significantly higher than that in the non-HTG-AP group. This study also provided strong support for the “lipotoxicity-inflammation” cycle hypothesis in the pathophysiology of HTG-AP. The latest research has further found that hyperlipidemia can destroy the intestinal barrier by down-regulating the expression of glutathione S-transferase pi (GSTpi) in the intestine. This process activates the NOD-like receptor thermal protein domain associated protein 3 (NLRP3) inflammasome, thereby exacerbating the systemic inflammatory response and pancreatic injury in HTG-AP (32). The above mechanisms suggest that early intervention in hyperlipidemia may help to block this vicious cycle.

The combination of strong inflammatory response and severe metabolic disorders makes HTG-AP patients more prone to moderate to severe acute pancreatitis. Compared with other causes, HTG-AP progresses faster and has a higher incidence of complications. Multiple clinical studies have confirmed that HTG-AP patients have a higher proportion of systemic inflammatory response syndrome (SIRS), multiple organ dysfunction syndrome (MODS), and pancreatic necrosis (4, 33). Our data also showed that patients with HTG-AP had a significantly higher ICU admission rate than those with non-HTG-AP (*P* = 0.029). In addition, there was no significant difference in hospitalization days between the two groups, which may be related to the sample size.

This study has some limitations. Firstly, this was a single-center study with a relatively small sample size. Secondly, the follow-up duration for patients with PPDM-A was limited. Thirdly, due to the predefined design of our prospective protocol, formal severity scoring systems (e.g., BISAP, Ranson, Glasgow) and several laboratory markers including HbA1c and lactate were not prespecified for collection. In addition, data regarding post-discharge recurrence, local complications and mortality were not systematically recorded. Furthermore, this prospective study was conducted exclusively in an adult hospital; therefore, patients under 18 years of age were not enrolled. Future multi-center, large-sample prospective studies with prolonged follow-up and inclusion of pediatric cohorts are warranted to validate our findings. Meanwhile, further in-depth investigations into the interactive mechanisms between metabolic disorders and inflammatory responses in HTG-AP are required, which may provide novel therapeutic targets for the precise management of HTG-AP.

In short, there are significant differences in clinical characteristics between HTG-AP and other causes of AP. HTG-AP patients showed younger onset age, more significant metabolic disorders and more severe systemic inflammatory response. HTG-AP is more likely to progress to moderate to severe pancreatitis and is associated with an increased long-term risk of new-onset diabetes. Early identification of high-risk features related to metabolic disorders and inflammatory responses, along with timely lipid-lowering and anti-inflammatory interventions, may improve both the acute prognosis and long-term outcomes in patients with HTG-AP.

## Data Availability

The original contributions presented in the study are included in the article/supplementary material. Further inquiries can be directed to the corresponding author.
